# Lieut.-Colonel Simpson Powell

**Published:** 1911-04-15

**Authors:** 


Lieut.-Colonel Simpson Powell, M.D., R.A.M.C., who died at Rangoon, aged 53, received his medical education at King's College, London. He took his M.B. degree (with honours) at Durham in 1883, and graduated M.D. in 1896. He entered the Army Medical Service in 1885, and served in India until 1892. Later in England he was appointed medical officer to the Grenadier Guards for five years. In 1897 he again went to India and in 1904 to Tientsin as senior medical officer. On returning to England he was in charge of the Louise Margaret Hospital, Aldershot. He was gazetted lieutenant-colonel in 1905. In 1908 he again went out to India, and the following year to Burma.

